# DNS for IoT: A Survey

**DOI:** 10.3390/s23094473

**Published:** 2023-05-04

**Authors:** Ibrahim Ayoub, Sandoche Balakrichenan, Kinda Khawam, Benoît Ampeau

**Affiliations:** 1 Association françAise Pour le Nommage Internet en Coopération (Afnic), 1 Rue Stephenson, 78180 Montigny-le-Bretonneux, France; sandoche.balakrichenan@afnic.fr (S.B.); benoit.ampeau@afnic.fr (B.A.); 2 École Doctorale Sciences et Technologies de l’Information et de la Communication (ED STIC), Université Paris-Saclay, 3 Rue Joliot Curie, 91190 Gif-sur-Yvette, France; 3 Laboratoire DAVID, Université de Versailles St-Quentin, 45 Avenue des États-Unis, 78035 Versailles, France; kinda.khawam@uvsq.fr

**Keywords:** DNS, DNS security, IoT, IoT challenges

## Abstract

The Internet of Things (IoT) is paving the way to becoming necessary in numerous aspects of people’s lives. IoT is becoming integrated in many domains, such as medical, industrial, and personal. Recent years have witnessed the creation of many IoT technologies that differ not only in their applications and use cases but also in standards. The absence of universally accepted standards and the variety of technologies are only some challenges the IoT market faces. Other challenges include the constrained nature of most IoT devices, the diverse identification schemes, the inadequate security mechanisms, and the lack of interoperability between different technologies. The Domain Name System (DNS) persisted throughout the years as the Internet’s naming service and accumulated more trust from users with the introduction of its security extensions. DNS could be utilized to address some of the challenges the IoT market faces. However, using DNS for IoT applications might jeopardize DNS infrastructure. In this survey, we study the coexistence of DNS and IoT. We define IoT, present its architecture and discuss its main challenges. We then introduce DNS and its function; we discuss its security and privacy drawbacks and the extensions standardized to address them. We further discuss the uses of DNS in IoT environments to address some of IoT’s challenges and the impact these uses might have on DNS.

## 1. Introduction

Like other breakthrough technologies, the Internet of Things (IoT) shapes our world and how we perceive it. If the average user were asked 20 years ago about her first thought when hearing the term *connected device*, the answer, if any, would probably be the personal computer. Cars, refrigerators, pets, and other daily-use objects were never thought to one day need a network connection. Even phones were only known as voice-call devices and had limited functionality besides that. Currently, however, the definition of connected devices has vastly changed. Personal computers are no longer the only ones with network connectivity; they have become only one of many categories of connected devices. One of the catalysts for this change was the introduction of smartphones, the devices that redefined our relationship with the Internet. Smartphone users are no longer required to be seated at a desk to access the Internet. Pocket-size mobile phones became the primary interface with the network, and users could hardly imagine being disconnected as many services became available online. After that, the appreciation of connected devices increased, and the IoT industry boomed. The number of connected devices and their functions and underlying standards increased. Currently, with the deployment of 5G, IoT technologies are expected to benefit immensely. 5G offers faster data rates, lower latency, and greater network capacity. Sensor networks are already reaping huge benefits from the reliability of 5G [1,2]. Consequently, many regular devices typically considered isolated are now connected.

**Figure 1:**
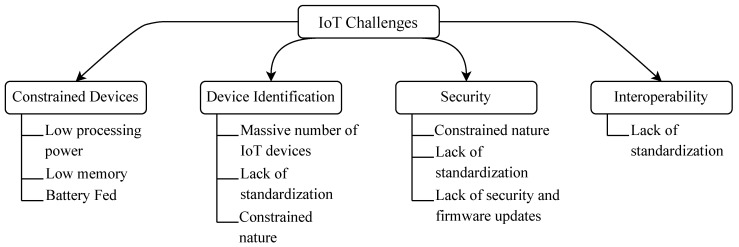
Taxonomy of challenges Facing IoT.

The average number of devices connected per household in the United States rose from 11 in 2019 to 25 in 2021 [3]. The predictions about the global number of IoT devices in the future vary anywhere between 25 billion [4] and 125 billion devices [5] in 2030, with total revenue of USD 1 trillion [6] to 1.5 trillion [7]. These predictions illustrate the significance of IoT, which will be increasingly palpable with time. Nevertheless, despite their omnipresence, IoT technologies face some challenges. Figure 1 presents the taxonomy of challenges facing IoT technologies.

The **first challenge** is that most IoT devices are constrained. Devices of this kind are designed to perform specific jobs, such as measuring temperature or detecting motion. These tasks do not require significant resources, and manufacturers of such devices aim to mass-produce while keeping the prices low. Therefore, constrained devices of this kind have limited memory and processing power and are usually battery-powered. The constrained nature of IoT devices complicates their management. Mechanisms commonly used on the Internet today for regular, more powerful devices cannot be directly used with constrained IoT devices. These mechanisms, for example, include encryption and decryption of data and receiving software updates or security patches. Such mechanisms, therefore, must be redeveloped to suit the less powerful IoT devices.

The **second challenge** lies in the identification of these devices. IoT or not, any device connected to a network needs an identifier. The most commonly used identifier on the Internet is the IP address. Identifiers serve at least one primary purpose: the data sender uses them to specify the exact recipient. On the other hand, the recipient can use them to verify the source of the data or to determine whether the data are intended for them. Identifiers must be unique in the scope they are used in to identify each device uniquely. The massive number of IoT devices, lack of standardization, and constrained nature impede finding a global identification scheme similar to IP addresses. The lack of global identification schemes, or at least large-scale ones, makes it challenging to manage and track the large number of IoT devices, leading to potential security vulnerabilities and interoperability issues.

The **third challenge** is security. Security of exchanged data is vital in any communication, and IoT communications are no exception. However, contrary to regular Internet communications, where security and privacy mechanisms are generally standardized and trustworthy, IoT technologies suffer from security drawbacks. These devices have a relatively large attack surface, making them vulnerable to several cyber attacks. IoT devices, for example, are the primary target of attackers who aim to launch Denial of Service (DoS) attacks, as these devices are easily compromised. The constrained nature of IoT devices prevents them from using up-to-date security mechanisms as they require more memory and processing power resources than these devices have. In addition, the lack of standardization also impairs security since every manufacturer uses a proprietary security mechanism that is predominantly incompatible with other manufacturers. Lastly, these devices are usually not adequately maintained regarding software updates, especially security patches.

Lastly, the **fourth challenge** we identify is the lack of interoperability between IoT manufacturers. The lack of global standards provokes heterogeneity between different IoT technologies. For example, a motion sensor from one manufacturer would not be able to communicate with a motion sensor from a different manufacturer or with the backend server of that sensor, even if they belong to the same network. Moreover, devices from one network would be unable to communicate with devices from different networks if these networks are based on different IoT technologies. Heterogeneity arises in identification, data formatting, and security mechanisms used, among other things. This causes the IoT environment to become vertically divided into separate silos where different technologies cannot communicate. This lack of interoperability disperses research and development efforts since a breakthrough in one technology is, with high probability, not useful to others. Interoperability between different IoT technologies is necessary for globally standardized IoT.

Given the spread of IoT technologies today and the projections of further large-scale deployments of its technologies, identifying and addressing its challenges is a must. Of course, having one remedy for all IoT drawbacks is not attainable, but it is possible today, with tools already available to ameliorate IoT environments and their user experience. So, instead of developing new ones, it would be meaningful and efficient to consider tools and standards we currently use on the Internet and adapt them to constrained IoT to address its challenges.

A service that stood the test of time as one of the cornerstones of the Internet is the Domain Name System (DNS). The DNS is the Internet’s naming service that has been essential for the Internet since its inception in 1987 [8]. The job the DNS does, along with its efficiency and robustness, rendered it an essential service. Drawbacks of the DNS, which mostly revolve around its not so security-oriented original design [9], are being addressed by many initiatives, which resulted in several protocol evolutions to make DNS more secure. For the billions of connected devices forming the IoT, DNS is also a necessary tool. Even the simplest IoT devices, such as thermometers or motion sensors, might need to use DNS to contact their backend servers [10]. IoT technologies could also utilize the DNS in other manners. For example, the functions of DNS and its distributed nature make it an ideal candidate for IoT naming and identification. Some proprietary IoT naming schemes are already using it [11]. Moreover, its recent security extensions help make IoT communications more secure if adapted to constrained devices.

However, having discussed the former points about DNS and IoT and how DNS could be useful, the possible repercussions for DNS should be noticed. According to the projections discussed earlier, billions of IoT devices will be added yearly. This raises questions regarding the scalability and security of DNS. There have already been some worrisome impacts on DNS due to IoT, such as the infamous Mirai attack [12,13].

In this work, we explore and examine the challenges IoT environments face. We primarily focus on challenges due to the constrained nature of IoT devices and challenges related to the identification of these devices, security of IoT communications, and interoperability between different IoT technologies. We elaborate on each challenge and investigate how DNS is being used to address some of these challenges. We then illustrate the effects this usage might have on the DNS infrastructure. Overall, this study aims to understand better IoT challenges and the benefits DNS might have to address these challenges. This work can provide valuable insights for IoT researchers, manufacturers, and policymakers of IoT technologies to create a safer, more reliable IoT ecosystem.

## 2. Related Work

Upon conducting our literature review, we noticed a lack of comprehensive surveys that address IoT challenges and how DNS could be used to address them. Even though several surveys touched on the subject by studying some uses of DNS with IoT, they were not comprehensive regarding the addressed challenges. Instead, most of these surveys dealt with one of the IoT challenges, mainly the security challenge, and studied how DNS could address that. Other surveys discuss IoT’s impact on the DNS infrastructure without a detailed discussion of the benefits IoT might receive from using DNS. Our survey, on the other hand, is more comprehensive. It introduces the concept of IoT, details IoT’s challenges, and then explains the functions of DNS and how DNS can be used to address these challenges. The survey ends with a discussion about the impact of IoT on the DNS infrastructure. In this section, we discuss the most notable surveys we found. We highlight their contribution and what they lack compared to our work.

The survey in [14] discusses how DNS is to be used, by IoT devices or intermediary devices such as gateways, to communicate with backend servers. The survey discusses the possible advantages IoT might gain from using DNS. These advantages include the security gains from DNS security extensions and device transparency gains from monitoring DNS-using IoT devices. However, rather than moving on to more IoT challenges that DNS solves, the work in [14] studies the risks to the DNS caused by using it in IoT applications. The main risk lies in the large number of IoT devices that could generate DDoS attacks against DNS either intentionally due to malware or unintentionally due to coding errors that could be encountered at the IoT scale. The work in [15] is also a survey regarding the uses of DNS in IoT environments. The effects of IoT on DNS are also discussed. The authors, however, did not explore the full potential of DNS as a tool to address DNS challenges. Other surveys discuss the challenges encountered in IoT environments. The work in [16] is a comprehensive survey about the possible security threats on IoT. The authors adopted a three-layer model and detailed the possible security threats on each layer. The two surveys in [17,18] are also about IoT security threats and possible solutions. However, Refs. [16,17,18] only discuss the security aspect of IoT challenges. Some works explored IoT when deployed in specific domains. The survey in [19] is about consumer IoT device classification using different Machine Learning-based methods. The study provides recommendations for creating the best possible home IoT environment. The survey in [20] discusses the uses of IoT in the maritime industry, while Ref. [21] is a literature review about IoT healthcare applications and the security and privacy requirements of IoT when used in healthcare. The surveys in [19,20,21], however, are only concerned with IoT implementations in specific domains and deal only with security challenges of such implementations. The work in [22] studies industrial IoT’s possible identity resolution systems. The paper presents a general framework for reviewing identity resolution systems. In [23], the authors studied the IoT identification techniques and the Manufacturer Usage Description (MUD) standard used to reduce the IoT attack surface. The paper studies the security of infrastructure instead of the security of IoT data. The authors in [24] presented three methods to detect IoT devices on the Internet, namely an IP-based method that detects servers that IoT devices contact from Internet flows, a DNS-based method that detects the names of such servers from DNS queries, and a certificate-based method that detects HTTPS-accessible IoT devices by inspecting their TLS certificates.

## 3. Survey Overview and Significance

IoT is expected to touch many aspects of daily life. Many objects connected today, like pets, cars, and home appliances, were never thought to one day need a network connection. This tells us that many objects we see today as isolated and needing no network connectivity will also be connected in the future. This is likely as IoT predictions are predominantly optimistic, and larger-scale future deployments are expected. These optimistic predictions demand a closer look at the present state of IoT to ensure that IoT growth will be safe and beneficial to all stakeholders. Having more connected devices means, on the one hand, more load on network infrastructure and, on the other hand, more personal data entrusted to these devices. Hence, it is pivotal to study the IoT environment’s current challenges and elaborate on possible solutions. Addressing these challenges will allow a larger adoption of IoT technologies. It is important to highlight these challenges, study their effect on limiting IoT today, and study possible solutions. When thinking about solutions for IoT technologies, it is essential to find solutions that do not themselves create further challenges. For example, focusing on name resolution for a single IoT technology that is not interoperable with other technologies is counterproductive since IoT as a technological concept will not benefit from that solution, only that specific technology for which the solution was designed. Hence, considering solutions encompassing as many IoT technologies as possible is of great benefit and importance. DNS could play a significant role in addressing current IoT networks’ challenges. DNS, the domain name system that converts domain names to IP addresses, could benefit IoT by resolving the domain names of IoT devices or their backend servers. Moreover, DNS’s security extension could play a role in securing IoT DNS traffic. However, using DNS in IoT environments could be rewarding for IoT but could simultaneously negatively impact the DNS infrastructure. The work in this survey aims to explore this coupling between IoT and DNS. The research questions we aim to answer are the following:

1. What are the main challenges facing the implementation and adoption of IoT on larger scales?
2. How can the Domain Name System be used to address these challenges to facilitate the growth of IoT safely and cooperatively?
3. What impact could the large-scale implementation of IoT have on DNS?

**Figure 2:**
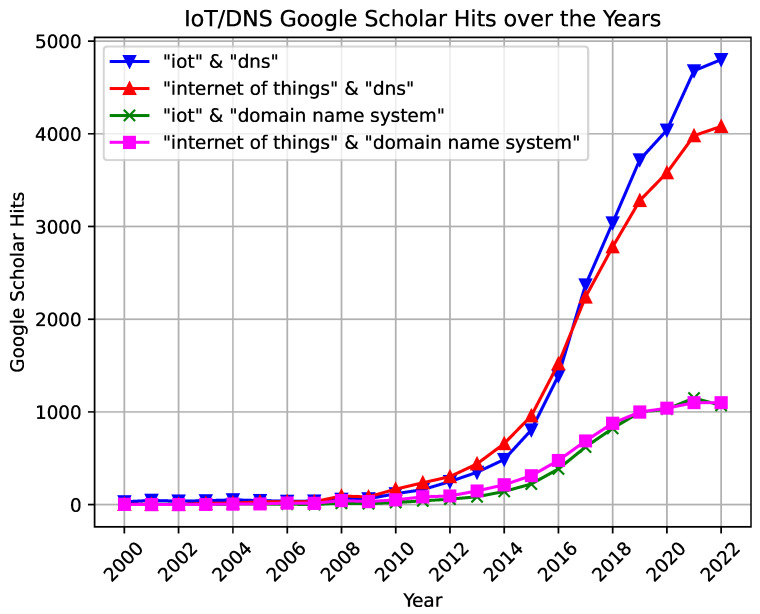
IoT/DNS Google Scholar hits between the years 2000 and 2022.

Upon researching the topic, we noticed an increased interest in IoT and DNS, specifically in articles mentioning the two terms. Figure 2 is a visualization of this interest, and it shows the number of hits on Google Scholar for articles mentioning IoT and DNS between the years 2000 and 2022. The figure shows an upward trend in results mentioning IoT and DNS. This is merely an inconclusive indicator but shows that an association between the two terms is getting stronger. We aim to form a solid starting point for research that tackles the different IoT challenges by using DNS as a significant contributor to alleviate these challenges while minding IoT’s impact on its infrastructure.

Our contributions in this work:

- We introduce IoT, its applications, and its main challenges.
- We explain the function of DNS, its security drawbacks, and the most important DNS security protocols and extensions.
- We perform a comprehensive literature review to investigate utilizing DNS to address the various challenges of IoT environments. To the best of our knowledge, a comprehensive survey of this nature has not been previously carried out. Hence, we perform a literature review about DNS usage in IoT environments to address the various challenges.

## 4. Method

Our survey covers three main topics. Firstly, it introduces IoT, its applications, and its challenges. Secondly, it introduces DNS, its security drawbacks, and the relevant security extensions and protocols. Finally, it studies how DNS is used in the literature to serve IoT environments and examines how IoT might affect the DNS infrastructure.

We searched for relevant articles in various databases, including IEEE Xplore, Google Scholar, ACM Digital Library, SpringerLink, and ScienceDirect, to investigate these topics. We included articles published after 2018 to ensure that our work is up-to-date. However, we also included a few articles published before 2018 if they had a significant contribution and reinforced our understanding of the topics. We included scientific conference papers, research papers, surveys, and technical reports. We also studied and cited several IEFT Requests for Comments (RFCs), as these documents are the standards in the industry. We considered relevant RFCs regardless of their publication date. The keywords we used include: DNS Security, DNS and IoT, DNS IoT Survey, IoT Challenges, and IoT Security.

We screened the collected papers based on their titles, abstracts, introductions, and conclusions. We included articles that covered one of our three main topics: DNS and DNS security, IoT and IoT challenges, and the coexistence of DNS and IoT. In addition, we added relevant articles from the reference lists of the included articles and from articles that cite them. We excluded articles not written in English or that diverted greatly from our survey’s main topics.

We finally studied in detail the articles we obtained from the initial screening. The articles that were eventually included in our survey had to discuss explicitly at least one of the following topics:

- The concepts of IoT, its architecture, and applications.
- IoT’s challenges, including the constrained nature of IoT devices, IoT identification, interoperability between IoT technologies, and IoT security.
- DNS function, security and privacy drawbacks, and security and privacy extensions and protocols.
- The use of DNS in IoT environments to address at least one of the IoT challenges mentioned above.
- The effect IoT might have on DNS. Here we considered negative effects.

The list of abbreviations used in this paper is presented in Table 1.

**Figure 3:**
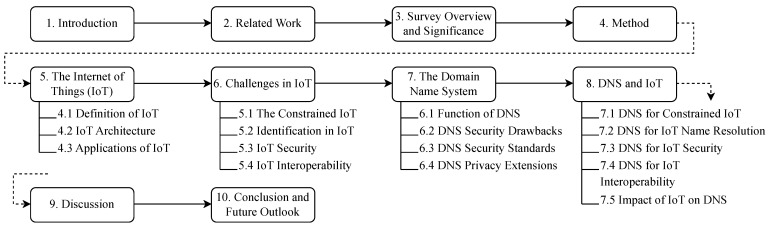
Contents of the Survey.

The work is divided as follows. Section 5 introduces IoT and its applications. Section 6 lists IoT challenges. Section 7 introduces DNS, its functions, and its security drawbacks. Furthermore, we explain DNS’s most common security extensions, which could serve both regular and IoT-connected devices. The literature is not rich with surveys about using DNS in IoT environments, even though we found numerous articles where DNS is purposefully used to address IoT challenges. Accordingly, Section 8 is a literature review of DNS-related IoT applications. The impact IoT might have on DNS is studied as well. In Section 9, we discuss our findings. We finalize with a conclusion in Section 10. The contents of the survey are detailed in Figure 3.

## 5. The Internet of Things (IoT)

Even though the Internet of Things (IoT) has been around for a while (the term Internet of Things was coined in 1999 by Kevin Ashton [25]), it is not until recently that it became an appealing topic in industry and research. Recent years have witnessed a rapidly growing IoT integration in many industries and research domains. Unlike Internet devices which are predominantly IP-based and use the TCP/IP protocol suite, IoT is not a single technology and is not governed by a single organization. It is a general term encompassing different technologies with high diversity in terms of communication protocols, data representation, and transmission technologies. The technologies are numerous and include, but are not limited to, Narrowband IoT (NB_IoT) [26,27], Bluetooth Low Energy (BLE) [28], Sigfox [29], Zigbee [30], and LoRaWAN [31].

### 5.1. Definition of IoT

A standard definition of IoT has yet to be agreed upon, but we can infer a definition from its applications. It is the network created by connecting physical or virtual objects to the Internet, including many objects that were not traditionally considered to require Internet connectivity. The ITU Telecommunication Standardization Sector (ITU-T) defines IoT in Y4000: Overview of the Internet of things [32] as “a global infrastructure for the information society, enabling advanced services by interconnecting (physical and virtual) things based on existing and evolving interoperable information and communication technologies (ICT)”.

The devices in an IoT network, again, according to [32], could be:

- Data carriers: These are usually static, such as barcodes, and attached to physical things.
- Data-carrying devices: These devices might have the information stored in them altered by a data-capturing device, e.g., Radio-frequency identification (RFID).
- Data-capturing devices: These devices can read from or write to a physical thing. The physical thing could be a data carrier or a data-carrying device.
- Sensing and actuating devices: This category includes sensors that can interact with their environment, gather data and measurements, and send them over the network for processing. This category also includes actuators that can perform operations in their environment based on information received over the network or their measurement of the environment around them.
- General devices: These include industrial machinery, home appliances, personal computers, and mobile phones that have embedded processing and wired or wireless communication capabilities.

### 5.2. IoT Architecture

**Figure 4:**
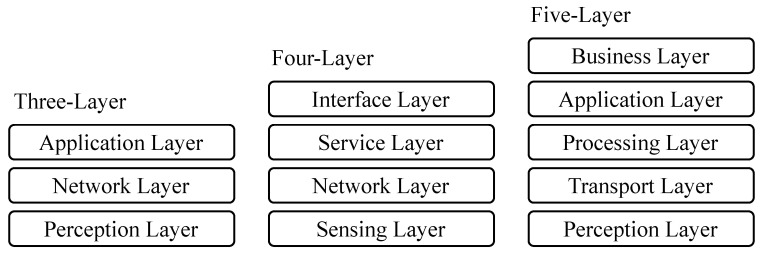
IoT architectures.

As with its definition, IoT has many standard architectures. The most notable are the three-layer [33,34], four-layer [35], also referred to as Service-Oriented Architecture (SOA), and five-layer [34] architectures. See Figure 4.

#### 5.2.1. Three-Layer Architecture

- **Perception Layer:** This layer contains devices like sensors, actuators, barcodes, and RFID tags. It is mainly the layer that interacts with the environment to collect data to be sent to the upper layers.
- **Network Layer:** This layer receives data from the perception layer. It includes the network infrastructure that transfers data between the Perception and Application layers.
- **Application Layer:** This layer processes and analyzes data passed through the network layer.

#### 5.2.2. Four-Layer (SOA) Architecture

- **Sensing Layer:** This layer is similar to the perception layer in the three-layer model. It contains sensing tools to perform the measurements in these devices’ environments.
- **Network Layer:** This layer is the network infrastructure (wired or wireless) that ensures connectivity between things among themselves and between things and their backend.
- **Service Layer:** This layer stores and processes information. Services required by users are created and managed here.
- **Interface Layer:** This layer defines interaction rules between users and devices. It also attempts to solve compatibility issues between devices from different vendors that follow different standards.

#### 5.2.3. Five-Layer Architecture

- **Perception Layer:** This layer is similar to the Perception Layer in the three-layer architecture.
- **Transport Layer:** Also referred to as the network layer. This layer receives data from the perception layer and passes them to the processing layer.
- **Processing Layer:** This layer processes and analyzes data passed through the network layer.
- **Application Layer:** Data from the processing layer are used here for IoT applications.
- **Business Layer:** This layer contains the management of the IoT system.

### 5.3. Applications of IoT

Applications of IoT are numerous and diverse. Smart cities use IoT devices to monitor traffic conditions [36], predict pollution levels [37], and provide smart parking [38]. Furthermore, cities deploy IoT networks for security purposes such as asset monitoring [39] and identification [40]. The agriculture industry benefits from IoT for produce distribution [41], supply chain management [42], and overall smart agriculture [43]. IoT plays a significant role in the health sector and is commonly known as the Internet of Medical Things (IoMT). IoMT helps improve the quality of life while decreasing the pressure on the medical system by allowing patients to self-diagnose when possible or to obtain a healthcare professional’s recommendation from a distance [44,45]. Care for the elderly [44], monitoring the state of mind [46], physical activity [47], and even eating habits [48] are a few examples of what IoMT has to offer in terms of ameliorating the healthcare system. The digital transformation also found its way to different industries, leading to what is referred to as the fourth industrial revolution (Industry 4.0) with the Industrial Internet of Things (IIoT) [49]. In addition, IoT proved to be helpful in emergencies where communication with the individuals at risk is of utmost importance [50,51,52,53]. Finally, in light of the COVID-19 pandemic, IoT has been used to help trace, detect, and consequently mitigate the spread of the virus [54,55].

The predictions that IoT will grow significantly are already starting to materialize. The diverse domains discussed above that are driven forward by incorporating IoT in their functioning corroborate these predictions. Therefore, it is evident that IoT technologies have to be in their best form to connect everything securely. This highlights the importance of pinpointing the shortcomings of IoT and the challenges its various technologies face. Accordingly, we explore today’s IoT challenges in Section 6.

## 6. Challenges in IoT

The challenges facing IoT could be divided into the following: the constrained nature of IoT devices, their identification, security, and the interoperability between different IoT technologies. We introduce these challenges in the following subsections.

### 6.1. The Constrained IoT

A considerable fraction of IoT devices is constrained. According to RFC 7228 [56], titled ‘Terminology for Constrained-Node Networks’, constrained devices are those that have:

- Limited ROM\Flash, leading to limitations on the maximum code complexity;
- Limited RAM, leading to constraints on the buffer sizes;
- Limited processing power;
- Batteries as sources of power;
- Constraints on user interface and accessibility in deployment.

Table 2 shows the classes of constrained devices according to their data and code sizes. Class 0 devices are ultra-constrained and need intermediary devices to communicate with designated servers. Class 1, on the other hand, could use protocols explicitly designed for constrained devices, such as Constrained Application Protocol (CoAP) over UDP [57]. Finally, Class 2 devices, which are more powerful, could use protocols used by regular devices on the Internet and benefit from lightweight specifically-designed protocols.

Such devices usually engage in gathering data from the real world. The data collected by these devices are generally sent to one or more servers on the network for processing. Due to the constraints mentioned above, designers of such devices are limited in choosing their operating systems or security mechanisms. The low processing power and low memory force the designers to abandon today’s well-maintained popular operating systems running on personal computers and servers. Instead, they go for operating systems that comply with the stringent constraints of IoT devices but need a better reputation regarding performance and code complexity, leading to a bad reputation in security. In addition, these devices’ power sources, mainly batteries that are expected to last for years, add further constraints. Consequently, such devices are only active for a short time, mostly use low bit rates, and usually lack user interfaces, making them harder to maintain and monitor.

### 6.2. Identification in IoT

Whenever an entity needs to connect to a network, it will need identification. People use these identifiers daily, but it may have become second nature that they overlook them. The mobile phone number is one of the most widespread identification methods for devices connected to networks. It provides a unique global identifier for every user. Thanks to its uniqueness, a user can initiate calls and receive calls from any other mobile phone number. This uniqueness guarantees that only the intended user is contacted and not any other user on the network. Regardless of the context where the identifier is used, its construction must follow an identification scheme. Identification schemes formulate the rules to be followed when creating and using an identifier. For example, domain names are identifiers used in the context of the Internet to identify resources. However, creating a domain name is not random but abides by rules and regulations, i.e., by the identification scheme mentioned in RFC 1035 [58].

The need for identification also applies to IoT devices. However, identification in IoT is not straightforward, and the hurdles stem from the interoperability challenge mentioned earlier. Different technologies use different identification schemes which are not interoperable. This further vertically divides the IoT environment since globally comprehensible identifiers are necessary for fixing interoperability problems.

The heterogeneity between IoT technologies and the lack of global standardization complicates discussing how identifiers should be, what properties they should have, or what requirements they should abide by. However, several vendor-neutral initiatives have tried to create a framework for IoT identifiers. These initiatives helped set up a taxonomy and requirements for IoT identifiers.

#### 6.2.1. Taxonomy of IoT Identifiers

The *EU–China Joint White Paper on Internet-of-Things Identification* issued by the EU–China IoT Advisory Group [59] and the *Identifiers in IoT* report issued by The Alliance for Internet of Things Innovation (AIoTI) [60] define a taxonomy for IoT identifiers. Table 3 presents this taxonomy with the function and use cases for each category.

#### 6.2.2. Requirements for IoT Identifiers

Table 4 lists the most common requirements for IoT identifiers suggested by AIOTI [60] and the ITU-T [61].

#### 6.2.3. IoT Identification Schemes

The identification schemes in IoT are diverse. Unlike machines using the TCP/IP protocol suite identified by unique IP addresses, IoT devices are defined by whichever identifier their manufacturer decides to use. We introduce some of the most known IoT identification schemes today in the following. We mainly focus on ones that are widely used in practice. We start with IP, which has been used as an identifier in 6LoWPAN. We follow that with the Digital Object Identifier, Electronic Product Code, and Object Identifier.

- **IP Addresses:** We start with the most common identifier, the Internet Protocol (IP) address. In the context of IoT, IP typically means IPv6 since the 32-bit IPv4 is already exhausted. On the other hand, the 128-bit IPv6 has a massive address space ($2^{128}$ addresses) and could theoretically accommodate existing and future IoT devices. RFC 4919 [62] defines how IPv6 is used in Low-Power Wireless Personal Area Networks (LoWPANs). The devices in LoWPANs conform with our definition of constrained IoT devices. According to [62], these devices conform with the IEEE 802.15.4-2003 standard and are characterized by short-range, low bit rate, low power, and limited computational power and memory. IPv6 is used in such networks as a unique identifier for each device within the scope of the network. Other than its large address space, IP is preferential because it has been around for a long time. Therefore, its standards and regulations are readily and freely available and well-known in addition to its existing infrastructure that includes management, diagnostic, and commissioning tools [62].
- **Digital Object Identifier:** The Digital Object Identifier (DOI) is defined in the ISO 26324:2012 standard [63]. It was initially developed by the International DOI Foundation (IDF), which three publishing institutes formed [64], and later standardized by ISO 26324:2012 [63]. Later, the DOI system became the go-to tool for identifying objects digitally. The word Digital in Digital Object Identifier refers to the identifier. DOIs are meant to be unique, persistent, and permanent digital identifiers for objects. Information about an object identified by a DOI can be retrieved upon resolving the DOI. DOIs are formed of two parts, prefix and suffix, separated by a “/” and with no maximum length. The prefix identifies a unique naming authority that is responsible for assigning DOIs. The suffix is a unique identifier of objects which, for interoperability purposes, could be an existing identifier. A typical DOI, for example, is 10.100/20.
  The Handle System [65,66] does the resolution of DOI. The Handle System is a set of distributed servers that allow the storage of handles that refer to digital objects. The system can efficiently and securely resolve the handles into information to locate and access the intended object. Additional services could be added, such as data confidentiality, data integrity, and non-repudiation.
- **The Electronic Product Code (EPC):** The EPC is a universal identifier for physical objects operated by GS1 [67]. It is used whenever an object needs to be tracked or identified. EPC is predominantly known as the ID used in Radio Frequency Identification (RFID). RFIDs act as data carriers holding the object’s EPC to which they are attached. However, this is not always the case. EPC is not exclusively used in RFID; the latter does not always contain an EPC. When stored on computer systems, EPCs are in the form of a Universal Resource Identifier (URI), often referred to as Pure Identity EPC URI. A typical Pure Identity EPC URI in Uniform Resource Names (URN) notation, for example, is
  $$\begin{array}{c} urn:epc:id:sgtin:0614141.112345.400 \end{array}$$
  On RIFD tags, and due to their memory limitations, EPCs are encoded in binary form. The resolution of EPC codes is accomplished using the Object Name Service (ONS) [11], which is based on the existing Domain Name System (DNS).
- **Object Identifier:** The Object Identifier (OID) [68] was developed jointly by ITU-T starting with ITU-T X.660 series [69], and ISO through ISO/IEC 9834-1:2012 [70]. The goal was to provide an unambiguous persistent name for objects. The OIDs are organized in a hierarchical tree structure. The top level is called the root; under it are nodes from which many arcs branch infinitely. An object’s name is the path from the root downwards until the node related to that object is reached. A typical OID in dot notation, for example, is
  $$\begin{array}{c} 1.2.840.113549.1 \end{array}$$
  and in URN notation
  $$\begin{array}{c} urn:oid:1.2.840.113549.1 \end{array}$$
  For resolution, the OID Resolution System (ORS) is a DNS-based system that accepts queries about OIDs and returns associated information. Various objects, big and small, could be identified using OIDs, such as countries, companies, X.509 certificates, standards, and Simple Network Management Protocol Management Information Bases (SNMP MIBs), to name a few.

### 6.3. IoT Security

RFC 8576 [71] titled ‘IoT Security: State of the Art and Challenges’ gives a general overview of the security challenges facing IoT environments. IoT security drawbacks are not due to a lack of security mechanisms but have roots in the devices’ design. The constrained IoT devices are meant to serve particular purposes, such as measuring temperature or detecting motion. Therefore, vendors of such devices seek to keep the designs simple and the prices low. So, most constrained IoT devices were designed without considering the security threats they may face. These facts prevent these devices from using modern security functions designed for more powerful ones. So, they either abandon security or use weak protocols and implementations [71]. In addition, and also due to the factors mentioned before, given their constrained nature and rapid development, some IoT devices do not receive necessary firmware updates as often as they should [72]. These firmware updates should be regular to avoid depriving these devices of possibly essential security updates. IoMT is one domain where security and privacy are paramount due to the sensitivity of the patient data. It is, however, vulnerable to the same attacks as other IoT devices [73,74,75].

**Figure 5:**
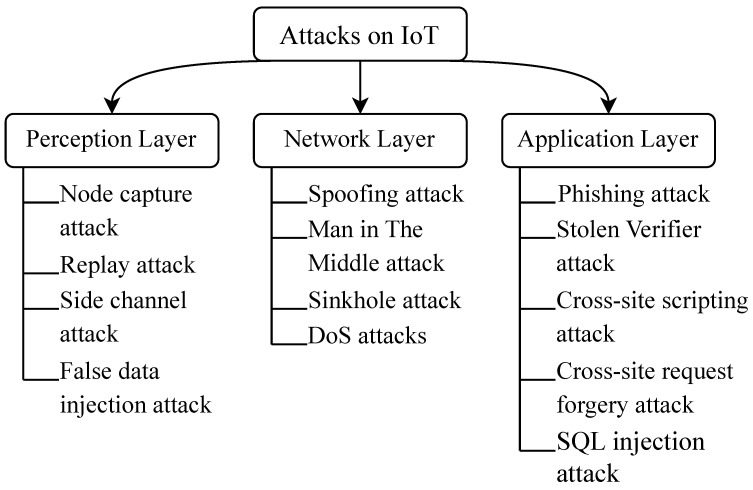
Attacks against IoT (three-layer architecture).

Figure 5 shows the classification of some IoT attacks [76,77] based on the three-layer model for IoT architecture.

- Perception Layer Attacks
  − Node capture attack: A physical attack against IoT nodes where the attacker captures the node and gains control over it. The attacker can then either impersonate the node, block incoming and outgoing traffic, or gain unauthorized access to the network associated with the node.
  − Replay attack: Replay attacks happen when the attacker captures a legitimate message destined for the device and saves it. The attacker later retransmits (replays) the same message to trick the devices about the sender’s identity. Replay attacks could allow attackers to access the network and control the devices.
  − Side-channel attack: Side-channel attacks occur due to unintentional device information leakage. This includes power consumption, acoustic emissions, or timing information. Using this information, an attacker might be able to, for example, guess the device’s key based on the power consumption during encryption or decryption.
  − False data injection attack: Occurs when attackers inject false data into devices. These attacks can be carried out manually on compromised devices, using Man-in-the-Middle or malware. False data injection attacks are particularly dangerous in critical applications such as healthcare.
- Network Layer Attacks
  − Spoofing attack: Occurs when an attacker impersonates legitimate devices or users on the network. This is achieved by altering data to make them look like they originated from other users or devices. Spoofing includes, for example, MAC spoofing, IP spoofing, or DNS spoofing.
  − Man in The Middle attack: Such attacks happen while data is transmitted between devices or between a device and its backend servers. The attacker intercepts the data and could sniff, alter, or block the communication.
  − Sinkhole attack: Occurs when attackers redirect IoT traffic to a compromised device or malicious server instead of the legitimate destination. This is accomplished by controlling the DNS or routing infrastructure.
  − DoS attack: Denial of Service (DoS) attacks occur when a large number of compromised devices are used to overwhelm a target server by sending a large number of requests. The target could be a DNS server or a regular web server.
- Application Layer Attacks
  − Phishing attack: Occurs when an attacker sends a malicious file or link to users of IoT devices posing as a legitimate entity such as a service provider or a manufacturer. Malware is installed upon clicking the link or opening the file, which might grant the attacker access to the network and control over its devices.
  − Stolen Verifier attack: Occurs when an attacker obtains a password or an authentication token that grants them access to the network and the devices connected to it. The attacker can then impersonate the users or devices of the network, leading to possible information theft or data corruption.
  − Cross-site scripting (XSS) attack: An injection attack where the attacker injects malicious code into a webpage or web application. This could be, for example, the web interface for managing IoT devices. As a result, the attacker can control the devices, steal sensitive information, or corrupt data.
  − Cross-site request forgery (CSRF) attack: Occurs when an attacker tricks a user into performing a malicious action on the webpage the user is already authenticated to. The attacker can then gain access to the network, steal or corrupt data, or control the devices connected to that network.
  − SQL injection attack: Occurs when an attacker inserts malicious SQL statements into an application’s input field. As a result, the attacker can corrupt, steal, or modify data. In the context of IoT, an IoT device with a web interface that accepts user input, such as a security camera with a login page, may be vulnerable to SQL injection attacks.

The facts above drove the research community to look into IoT vulnerabilities and the consequences such vulnerabilities may have on different security objectives if exploited by attackers [78,79,80]. IoT has benefited from the development of new technologies, such as Blockchain [76,81,82], and the advances that leverage Machine Learning (ML) [83,84] to improve security and mitigate attacks.

### 6.4. IoT Interoperability

In IoT, each vendor has its own infrastructure, devices, Application Programming Interfaces (APIs), and data formats [85]. This causes an interoperability problem between different technologies. Interoperability is regarded by the ITU-T as one of its high-level requirements for IoT, stating that “interoperability is essential among heterogeneous and distributed systems for the provision and consumption of a variety of information and services” [32].

RFC 8477 [86] attributes the lack of interoperability to the lack of an encoding-independent standardization and the link between the data formats and the technology that produces it. There is no shortage of IoT standards, but their abundance and the fact that they originate from numerous organizations [87] make achieving interoperability harder. Heterogeneity could be seen as device-level heterogeneity due to the various technologies and protocols used in devices, data-level heterogeneity due to various formatting of data, and semantic heterogeneity related to how different technologies interpret data they receive from other technologies [88]. Semantic heterogeneity and achieving semantic interoperability have been studied extensively [88,89,90,91]. Due to the lack of standardization, IoT technologies send and receive data in proprietary forms. One IoT device could not understand the meaning (semantic) of what some other device sent if the latter belonged to a different technology. Solutions for semantic heterogeneity include using a middleware, ontology, and semantic web of technologies.

Several organizations issued proprietary standards in an attempt to facilitate interoperability. We present two of them: The Web of Things and OneM2M.

**The Web of Things:** The Web of Things (WoT) [92,93], created by the World Wide Web Consortium (W3C), is a concept that aims to enable interoperability between existing IoT technologies by connecting things in these networks to the web. WoT aims to preserve and complement existing IoT technologies rather than implement new ones. The main building blocks of the WoT are:

- Thing Description (TD): The TD is a central building block of WoT. It contains metadata describing the thing, a set of Interaction Affordances indicating how the thing can be used, schemas for the data exchanged with the thing for machine understandability, and web links to express the thing’s relation with other things or documents.
- Binding templates: These consist of reusable vocabulary and extensions to the TD format that enables a client to interact with diverse things exposing different protocols.
- Scripting API: An optional block that enables implementing the application logic of a thing using a common JavaScript API similar to web browser APIs.
- Security and Privacy Guidelines: Guidelines for secure implementation and configuration of things.

Human-assisted semantics translation would extend the WoT, allowing IoT devices to understand metadata from devices that use different semantics to increase semantic interoperability between heterogeneous devices further [94].

**OneM2M:** OneM2M [95] was established in 2012 as a partnership project between eight standardization bodies. The goal was to address the interoperability problem in IoT and machine-to-machine communications by promoting a global IoT standard. OneM2M does not propose a new standard but builds on the existing standards and aims to facilitate interoperability. This is achieved by defining a common middleware between IoT devices, communication networks, and IoT applications. This middleware service layer contains a suite of common service functions (CSFs) exposed to IoT devices and applications via RESTful APIs. CSFs are general-purpose services unrelated to specific IoT domains or technologies. They can be looked at as generic operating system tools that various IoT devices and applications could use. The claim is that OneM2M allows any IoT application to connect to any IoT device, facilitating interoperability between IoT silos.

The fact that IoT is developing fast and quickly becoming a need rather than a luxury requires its challenges to be addressed. These challenges, as discussed above, are numerous and, to some extent, intertwined. The root cause lies in the constrained nature of IoT devices. Whenever a device is made with such specifications, it is automatically deprived of many tools used by today’s computers and servers. Tools that deal, for example, with security, privacy, and identification are not compatible with constrained IoT. Another definite fact about IoT is the diversity due to the different technologies, each using its proprietary standards and tools. While developing new technologies is essential, the conspicuous lack of interoperability between these different technologies prevents IoT from becoming a global network. Furthermore, the lack of interoperability in IoT slows down the research backing it up, as different technologies disperse the research efforts, making progress slower and more individual to each technology rather than for the whole IoT environment.

The DNS is a valuable tool to address the challenges described in this section. However, DNS has its drawbacks, especially regarding security and privacy. In Section 7, we present its primary function, security and privacy drawbacks and the extensions developed to address them.

## 7. The Domain Name System

In the early stages of the Internet, a simple ‘HOSTS.txt’ file located on a single computer was responsible for the domain name-to-IP address translation. All hosts retrieved the ‘HOSTS.txt’ file via FTP. The bandwidth required to download this file was proportional to $N^{2}$ for a network of *N* hosts [8]. This solution worked properly when *N* was small, but the growth of the Internet mandated that a more scalable solution be found. The solution was the Domain Name System (DNS). The DNS is a distributed lookup service that is used to translate domain names (such as “www.afnic.fr (accessed on 24 April 2023)”) to IPv4 addresses (such as 192.134.5.37) and IPv6 addresses (such as 2001:67c:2218:302::51:231). The DNS acts as the Internet’s phonebook and ensures that communications on the Internet, such as email or simple web browsing, are easily and efficiently carried out.

Many standardization documents describe the functions of DNS. The IETF regularly publishes RFCs that define or update DNS functions. Since the first steps to naming hosts were laid out in 1982 by RFC 819 [96], which was followed by RFC 920 [97] that introduced the idea of domain names, DNS has undergone many updates, some of the major ones being RFC 1034 [8] and RFC 1035 [58], which were the blueprint of today’s DNS.

### 7.1. Function of DNS

**Figure 6:**
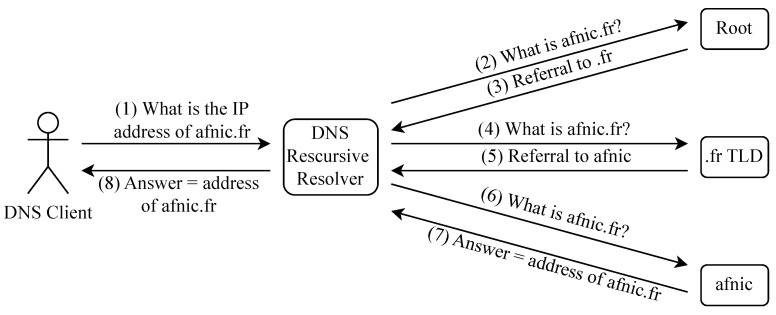
DNS resolution process.

The primary function of DNS is to map domain names to IP addresses. The domain names are usually given to resources on the Internet that users wish to access. The mapping process is referred to as domain name resolution. The DNS resolution process is depicted in Figure 6.

For example, a user that wishes to visit the website of Afnic (Afnic is the French Internet Registry) will type in their browser “www.afnic.fr (accessed on 24 April 2023)”. The first entity that receives the request, also known as the DNS query, is the stub resolver, which is usually part of the operating system. The stub resolver then transfers the query to the DNS recursive resolver. Recursive resolvers are the main interface between users and the DNS infrastructure. After receiving the query, the recursive resolver begins a hierarchical query/response process with the name servers of DNS. Name servers are DNS servers that map domain names to IP addresses. The set of all possible domain names and their associated IP addresses is known as the DNS namespace, and name servers that could return definitive answers to queries (i.e., answer with the IP address) about a subset of the DNS namespace are said to be authoritative over that subset.

The recursive resolver first sends the query to one of the root name servers. These servers are authoritative over Top-Level Domains (TLDs) such as .com and .fr. The root name server answers with a referral to the .fr name server. The recursive resolver then sends the request to the .fr name server, which is authoritative over .fr domain names. Hence, it can answer with a referral to Afnic’s name server, which is authoritative over “www.afnic.fr (accessed on 24 April 2023)”. Lastly, the recursive resolver sends the query to Afnic’s name server, which answers with the IP address of “www.afnic.fr (accessed on 24 April 2023)”. The recursive resolver then sends back the answer to the user.

### 7.2. DNS Security Drawbacks

When the DNS was first deployed, privacy and security were not considered [98]. Recently published RFC 9076 [98] titled ‘DNS Privacy Considerations’ studies the security (mainly privacy) drawbacks of DNS that end users should keep in mind. The drawbacks include sending the queries in plain text, which jeopardizes privacy, using UDP, whereas most security mechanisms are designed for TCP, sending the full QNAME (Query Name, i.e., the requested domain) at every stage of the resolving process even though it is not needed, sending unnecessary DNS requests due to resource prefetching and auto-completion, cache snooping, and the passive DNS data collection by powerful companies running public DNS servers like Google [99] and Cloudflare [100]. One cannot be sure that such valuable data are not sold to third-party companies for commercial purposes or abused for surveillance and spying. Finally, the Server Name Indication (SNI), the last plain-text part of a secured web connection, is worth mentioning. The SNI is used in the case of multi-hosting to identify the intended resource requested on the webserver. SNI is still sent without encryption, which exposes the browsing habits of users [101].

**Figure 7:**
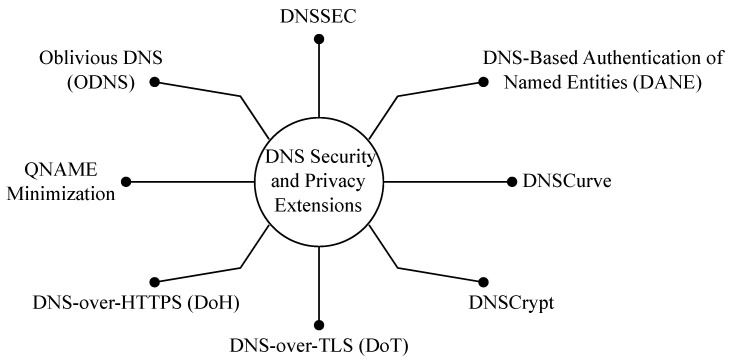
DNS security and privacy extensions.

We present next the most known DNS security and privacy standards and extensions. See Figure 7.

### 7.3. DNS Security Standards

If the DNS is to be used with IoT, as discussed in Section 8, it must ensure that communications to and from the constrained IoT devices are secure. We introduce the most known DNS security standards and extensions in what follows.

**Figure 8:**
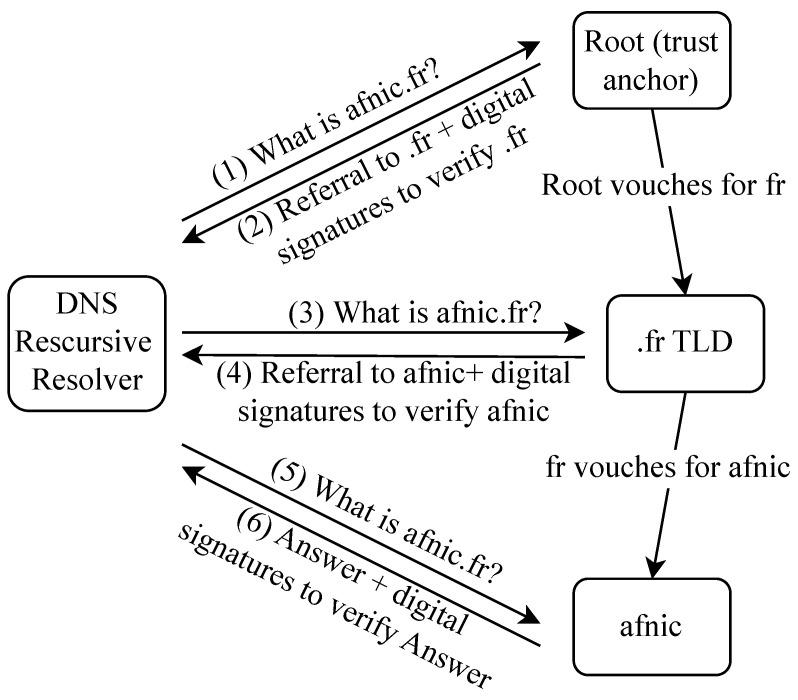
DNSSEC verification.

**DNSSEC:** DNSSEC [102] stands for DNS security extensions. DNSSEC guarantees the integrity (not privacy) of DNS responses that DNS clients receive using an authentication chain. The chain starts at the root servers, which are trusted by default. Going down, every level in the hierarchy vouches for the level below it. Cryptographic signatures are added to DNS resource records to ensure the DNS client that the record they receive came from the legitimate DNS server and that it has not been tampered with on the way. The DNSSEC mechanism is presented in Figure 8.

RFC 4033 [102] provides an introduction to DNS security and requirements, RFC 4034 [103] defines the Resource Records for the DNS Security Extensions, and RFC 4035 [104] is about the modifications required in the initial DNS protocols following the introduction of DNSSEC. RFC 4398 [105] discusses storing certificates in the DNS, RFC 5155 [106] presents DNSSEC hashed authenticated denial of existence, and RFC 6014 [107] explains how the cryptographic algorithm identifiers needed to implement DNSSEC are allocated in the Internet Assigned Numbers Authority (IANA) registries.

**DNS-Based Authentication of Named Entities (DANE):** DNS-Based Authentication of Named Entities (DANE), which was introduced in RFC 6698 [108], is a security protocol that allows domain name owners to associate digital certificates with their domain names to provide a way to authenticate and secure Internet communications. DANE uses DNSSEC to guarantee the integrity of certificates and associated domains.
**DNSCurve:** DNSCurve [109] was designed in 2009 to add link-level security to DNS using elliptic-curve cryptography. In particular, DNSCurve preserves confidentiality by encrypting DNS packets, protects the integrity of DNS responses by cryptographically authenticating them, and ensures availability by protecting against Denial of Service (DoS) attacks. DNSCurve uses 256-bit public and secret keys, 192-bit nonces, and 128-bit authenticators. DNS servers that use DNSCurve distribute their public keys by encoding them in regular nameserver (NS) records, ensuring that the public key distribution system is compatible with registries and name server software. On the other hand, clients share their public keys in the queries they send.
**DNSCrypt:** DNSCrypt [110] acts between DNS clients and DNS recursive resolvers. It uses cryptographic signatures to verify that responses from the resolvers are authentic and have not been tampered with on the way. Anonymized DNSCrypt [111] was proposed in 2019 as an extension to further secure DNS traffic by not allowing the server to see the client’s IP address.
**DNS-over-TLS:** DNS-over-TLS (DoT) [9,112] is one the few IETF-standardized DNS-securing protocols (the other notable one being DNS-over-HTTPS)). Instead of the traditional UDP, DoT uses TCP and provides packet authentication and confidentiality for DNS traffic between clients and resolvers. This is achieved using TLS. A TLS session is established on TCP port 853, and DNS data are exchanged over the secure channel.
**DNS-over-HTTPS:** DNS-over-HTTPS (DoH) [9,113] is the other IETF-standardized DNS-securing protocol. Its goal is also to preserve the integrity and confidentiality of DNS data. Moreover, as the name indicates, DoH uses HTTPS instead of benefiting only from the TLS handshake, as with DoT. The use of HTTPS allows a web application to use DNS securely. In DoH, any DNS query with its response is an HTTPS exchange. A client encodes the DNS request into an HTTP request using an HTTP GET or POST method.

### 7.4. DNS Privacy Extensions

This section describes other standards that introduced changes to the DNS protocol to preserve privacy. The most valuable information an adversary could retrieve from a compromised DNS query is about the source IP address and the requested domain or Query Name (QNAME). The source IP address may reveal the person or entity making that DNS request. Revealing the QNAME gives away the browsing habits of the query issuer and their email activity or at least which email exchange the query issuer is interacting with. Such knowledge could be exploited for commercial, political, or censorship purposes. The following paragraphs detail two approaches that altered the original DNS design to ensure confidentiality in DNS query resolution.

**QNAME Minimization:** One of the shortcomings of DNS is that the full QNAME and query type (QTYPE) are always sent during recursive DNS resolving, regardless of the stage of the resolving. Meanwhile, the full QNAME and QTYPE are only needed when the request reaches the authoritative name server of the domain requested. For example, when resolving “www.afnic.fr (accessed on 24 April 2023)”, the root name server receives a query with QNAME ‘www.afnic.fr’ even though it only needs to know .fr and does not need to know QTYPE. QNAME minimization [114,115] aims to allow resolvers to send minimum information at every stage of the resolution since the principle is “the less data you send out, the fewer privacy problems you have” [116]. Therefore, when sending queries to servers not authoritative for the requested domains, resolvers implementing QNAME minimization send a different QNAME to obscure the original one. Instead of the full QNAME, they send one level longer than what the name server is known to be authoritative for.
**Oblivious DNS (ODNS):** ODNS [117] addresses the fact that in any privacy setup, there should always exist a party that is trusted by default. The trusted party could be any entity from the ISP to large public DNS resolver companies. When a client sends DNS requests to their designated recursive DNS resolver, the resolver has complete access to the domains requested by the client and the client’s IP. Mostly, this recursive resolver is trusted not to abuse or share this data with third parties. The trust is almost baseless since one has no solid reason to trust their recursive resolver. ODNS aims to eliminate the need for that trust by preventing the recursive resolvers from associating between client identities and requested domains. ODNS uses the existing DNS infrastructure, which facilitates its deployment.

DNS was first designed with the goal of mapping between domain names and IP addresses. Several extensions and functionalities have been added to DNS since its inception. These extensions gave the DNS the needed maturity to become a reliable tool for any application that securely stores and retrieves information. DANE, for example, in which DNS is used as a complement or a total replacement of the certificate authorities for TLS handshakes, shows that DNS could play roles in areas previously thought to be out of its usual scope [118].

## 8. DNS and IoT

Section 7.3 and Section 7.4 demonstrated the ability of DNS to evolve. In particular, we explained how DNS evolved from being a basic name-to-address translation protocol to providing security and privacy for DNS traffic. In the following subsections, we explore the state-of-the-art works leveraging the DNS infrastructure and its protocols to tackle IoT challenges by providing seamless identification, interoperability, security, and privacy for IoT devices. We conclude the section with studies from the literature that evaluate the usage of DNS in IoT and the impact of the latter on DNS. Table 5 summarizes the use cases of DNS in IoT environments.

### 8.1. DNS for Constrained IoT

As described in Section 6.1, IoT devices are constrained in nature and have limited processing power, memory, and power resources. Since the DNS was not designed for constrained devices, its memory and bandwidth requirements are much more than what IoT can handle.

Even though most DNS responses fit in a 512-byte UDP packet [58], measurements between recursive resolvers and authoritative servers indicate that the network behavior is relatively uniform with IP packet sizes up to 1500 bytes [139]. DNSSEC operating with RSA signatures leads to significantly higher memory requirements [140]. Such large messages increase CPU usage and require high bandwidth.

QNAME minimization [114,115] and using elliptical curves [119] can considerably reduce the bandwidth and CPU usage. DNS over CoAP is another method that uses CoAP [57] for transport. CoAP allows for HTTP-like communication on constrained nodes [120]. DNS over Datagram Transport Layer Security (DTLS) [121] is based on TLS protocol and provides encryption for queries and responses between DNS clients and servers. It is more suited for constrained IoT scenarios that support low latency and loss-tolerant communication.

The constrained nature of most IoT devices will allow adopting DNS to solve many of its challenges. The DNS accounts for constrained devices through several extensions and could function according to their constraints.

### 8.2. DNS for IoT Name Resolution

Given the primary role of DNS on the Internet, which is to map domain names to IP addresses, it is expected to play a significant role in resolving IoT identifiers. Like regular Internet devices, IoT devices connected to the Internet will need identifiers and systems to resolve the identifiers into addresses. Addresses would eventually be used to query IoT devices for their readings, control and manage them, or be redirected to locations containing information about the device in question.

As explained in Section 6.2, the naming conventions for the IoT are numerous and, in most cases, incompatible. One possible way to solve heterogeneity in identification schemes is for a standardization organization to develop a globally unique naming convention and ask different stakeholders in the IoT space to use it. This could be done from a purely technical point of view. For example, the large IPv6 address space allows every IoT device to have a unique IPv6 address. However, having one global naming convention for all IoT devices will be nearly impossible. Industries like retail, automobile, and defense have proprietary naming conventions that they have used for a long time. Changing that would impact their infrastructure and operations considerably.

A more feasible alternative is to keep the existing naming conventions and develop the resolving process they use. Here is where DNS could be exploited. For example, DNS was used with ENUM (Electronic NUmber Mapping) to map telephone numbers to web addresses [141]. Moreover, there exist several DNS-based services such as the Object Name Service (ONS) [11] and the OID Resolution System (ORS) [68] that allow registration and resolution of unique identifiers for IoT devices. This shows the feasibility of DNS playing a more global role in name resolution for IoT, which will provide a familiar and robust solution for managing IoT devices and their namespace.

The work in [122] presents the problems different IoT platforms suffer from. The shortcomings of a particular system do not cause the problems, but they are caused rather because each technology has its naming scheme that cannot seamlessly connect to other IoT technologies and controls resolution over its defined namespace. The authors propose a new scheme for peer-to-peer equal name resolution. In the proposed method, names from various technologies are hashed into a string of bits. In addition to preserving privacy by one-way hashing of the name, it allows for resolution via DNS, which only requires adding a TLD (.iot). The authors in [123] studied the use of DNS architecture for IoT devoted to transport logistics. A hierarchical organization of DNS was presented that can scale globally by translating unique URIs to network addresses that can be used to extract information about the object of interest (status, location, etc.).

The global reach of DNS and its distributed nature were used in [124], where the existing DNS architecture was used to construct a search engine for the WoT, specifically a search engine for devices and their offered service. A TLD (.env, for example) is added. Users can then use a regular browser and look up, for example, service.location.env. The DNS resolving of service.location.env would return a list of devices offering the requested service at the specific location. Choosing a specific device requires only adding a level specifying the device requested to the name. The authors in [125] proposed a scheme to represent semantic metadata of IoT devices and encode those into domain names so that devices could be found by performing DNS queries. The paper also suggests ‘DNS as a source of IoT Data’, where DNS could be used to store TXT information about IoT devices.

DNS can help with device autoconfiguration. Autoconfiguration comes in handy in cases where the number of IoT devices is too large to be named individually. Autoconfiguration using DNS allows devices to name themselves and register themselves in their DNS zone. DNS Name Autoconfiguration (DNSNA) for IPv6-based IoT environments was proposed in [126,127]. DNSNA provides a global framework for IoT autoconfiguration, including defining DNS name formats for such devices, name generation, and registration of the generated DNS names. DNSNA uses IPv6 Neighbor Discovery Protocol (ND) to acquire the DNS search list through IPv6 Router Advertisement (RA) or DHCPv6. Once the DNS search list is acquired, the IoT device can generate its name using the DNS search list and its information. Authors in [128] extended DNSNA to IPv4 IoT devices by proposing DNSNAv4. IoT devices can register their DNS name using a DHCP server.

Moreover, the authors in [129] proposed IoTFinder, an IoT identification method using DNS traffic analysis. IoTFinder is a machine-learning-based multi-label classifier aiming to learn statistical DNS traffic fingerprints automatically. The authors in [130] presented IoTRoam, a roaming setup for IoT devices. The use case for IoTRoam was demonstrated with a LoRaWAN network where a Device could locate and join its dedicated Join Server with the help of DNS.

IoT is still far from having a global naming scheme with a global name resolution mechanism. A single solution to fit all the technologies is not realizable. However, the problem is still manageable. Some approaches leave the pre-existing naming schemes as is and work on an upper layer. Phone numbers, for example, are different in structure in each country but can interoperate globally using international codes. The diversity would not be eliminated, but it would be manageable. DNS can help many technologies interoperate while keeping their naming schemes intact. This, as discussed earlier, is already being done between individual technologies achieving peer-to-peer interoperability via DNS.

### 8.3. DNS for IoT Security

IoT presents several security risks to both consumers and businesses. As discussed in Section 6.3, the security risks in IoT environments are numerous. However, despite these risks, the security mechanisms currently deployed in IoT environments are inadequate and are usually proprietary as each IoT provider develops their own closed security solution. As a result, contrary to how security is set up on the regular Internet, there is no global security mechanism with known trust anchors for IoT technologies. Moreover, the constrained nature of IoT devices deprives them of security mechanisms currently used on the Internet that require more processing power and memory than most of these devices have. Since most IoT devices are highly constrained, bootstrapping trust, supporting secure communications, and guaranteeing privacy are significant challenges.

IoT data communication often needs a gateway to translate between the IoT device (e.g., sensors communicating via protocols such as Bluetooth or LoRa) and the end-points (e.g., cloud infrastructures using HTTP over IP) in the Internet core. One of the basic requirements in IoT is to control the terms under which an IoT device is allowed to onboard into the Internet core. Like regular devices on the Internet when joining a network, IoT devices must be authenticated when onboarding to a network. Such devices need an identifier and an authentication token to be admitted to the network. IoT technologies use different (predominantly incompatible) mechanisms when accepting new devices. On the one hand, these mechanisms are weak. On the other hand, the different mechanisms are incompatible, which worsens interoperability. DNS has been used to secure some aspects of IoT communications, whether with IoT devices joining and registering to their networks or subsequent communications.

The authors in [130] propose a Public Key Infrastructure (PKI) based on DNS for secure onboarding of IoT devices. The work in [131] built on DNSNA and added authentication. The obtained algorithm, Secure Domain Name System Name Autoconfiguration (SDNSNA) for IPv6 IoT devices, uses DNSNA for name generation and NFC-based authentication to authenticate and register their devices. The idea is that users can communicate with an authentication server using their smartphone, which can communicate with IoT devices using NFC. More on securing autoconfiguration and registration was carried out in [132] with Advanced Secure DNS name autoconfiguration with Authentication and Authorization for enterprise IoT network (ASDAI). The authors in [135] extended the EPC ONS based on DNS to dynamically support heterogeneous object code identification. The authors in [133] designed an IoT router that analyzes DNS traffic to detect if IoT devices consulted unknown DNS servers, which is usually done by malicious devices such as botnets. Botnets, which are compromised devices controlled by a Command-and-Control (C&C) server, typically use DNS to connect to that server [142,143]. DNS Filtering & Extraction Network System (D-FENS) was proposed in [134]. D-FENS sits between the client and the recursive DNS server and accepts DNS requests. It then uses a deep learning model to accurately detect and blacklist unreported malicious domains that IoT devices could connect to.

Several protocols and extensions were devised to secure the DNS and were discussed in sections IV-C and IV-D. These could benefit IoT devices using DNS. The benefits IoT could reap include security, privacy, and authentication. Moreover, as discussed earlier, DNS is no longer seen as only a mapping tool between IP addresses and domain names.

### 8.4. DNS for IoT Interoperability

The IoT infrastructure must incorporate different IoT connectivity technologies and hardware, software, identification, security, privacy, and resolution services. Multiple stakeholders provide these components and services. Beyond vertical integration, horizontal interoperability between devices and systems will be critical for an IoT network.

For different technologies, interoperability is the ability to communicate seamlessly. This includes exchanging and using data, services, or functionality, regardless of the underlying technology and standards. The issue of interoperability is one major drawback of IoT today. As discussed in Section 6.4, IoT technologies’ lack of interoperability is a significant drawback. This has caused the IoT environment to be fragmented into many incompatible technologies. These technologies develop and implement proprietary solutions and use communication protocols and data representation methods that few other technologies can understand. Thus, users and devices of one IoT technology are almost always bound to communicate with users and devices of the same technology. This fact transformed IoT into distant islands or silos, each having its standards.

DNS could play a role in alleviating some of IoT’s interoperability issues. Its robustness and distributed nature have already encouraged some organizations to resort to it when looking for IoT solutions.

The Object Name Service (ONS) [11] is EPC’s [67] resolution system that is based on DNS to resolve an EPC identifier to the location of the information associated with that identifier. The work in [135] suggests an enhancement to the ONS framework that enables it to handle heterogeneous object code identification. This technique shares similarities with established conventions such as OID [68] and DOI [63,64]. In [130], the authors laid out a PKI for IoT devices with an underlying technology based on DNS and its security extensions which aims to enable interoperability between heterogeneous IoT technologies. DNS-Based Service Discovery (DNS-SD) [144] demonstrates how DNS could be used to locate named entities. DNS-SD uses DNS Service records (SRV record) to locate services by specifying the specific service and the domain the service belongs to as service.domain. The client requesting the service receives a list of available services that fit the query as instance.service.domain, and then a service instance is chosen. DNS-SD is compatible with the standard unicast DNS and the DNS-like Multicast DNS (mDNS) [145]. DNS-SD has been used in [136] to develop an interoperable service discovery for IoT environments. Other works [137,138] also developed a solution for service discovery of resource-constrained devices based on mDNS/DNS-SD.

DNS plays a vital role in IoT interoperability. With its primary function as a naming service, along with its security extensions, DNS provides a reliable and secure way to map IoT device names to network addresses or information about these devices. This should encourage different IoT technologies to either consolidate their naming mechanisms or use services based on DNS to allow interoperability between the various naming standards. Doing this allows for building a cohesive Web of Things with distributed registries containing information about IoT devices and their services. DNS also supports dynamic updates, which allows for the automatic reconfiguration of device addresses, making it well-suited for dynamic IoT environments.

### 8.5. Impact of IoT on DNS

So far, we have encouraged using DNS to address many of the IoT environment’s challenges as we demonstrated how DNS could mitigate these challenges. However, we have also discussed the importance of DNS as one of the cornerstones of the Internet. The significance of DNS calls for caution when exposing this vital piece of the Internet’s infrastructure to IoT. The large number of IoT devices and the constrained nature of most devices could endanger DNS. IoT manufacturers usually give little to no thought to the security of their devices. These devices are seen as having specific functions and not needing much security. Moreover, the constrained nature of IoT devices prevents them from arming themselves with state-of-the-art security solutions used today on the Internet. In addition, constrained IoT devices such as sensors usually lack a user interface, so it is not easy for users to interact with them. This makes it hard for users to notice if their devices have been compromised and are being used to launch attacks.

In [146], some challenges facing DNS in IoT scenarios are explored. It mainly focuses on functionality, security, and availability problems. The work in [147] investigates whether the current DNS is ready for IoT. The authors lay out criteria that should be met before using DNS with IoT and analyze whether these criteria are met or not. The conclusions drawn could be summarized as follows:

- Security: Security is the main enabler of IoT, and although DNS security is enhanced, it remains too costly for constrained IoT devices.
- Mobility: An IoT naming service should support mobility and automatic name update. DNS is ready to provide the automatic name update, but since mobility was not accounted for when designing DNS, it lacks this feature for now.
- Infrastructure Independence: Name resolution should generally be independent of the underlying infrastructure. DNS could provide that with local link extensions and technologies such as cloud computing.
- Localization: All devices must be localizable and reachable. The authors argue that DNS is evolving to account for service deployment and name format localization.
- Efficiency: Efficiency is crucial for latency-sensitive IoT services. Efficiency remains a significant challenge for DNS because the DNS name resolution mechanism incurs delays due to the hierarchical delegation and unpredictable cache hits.

Even though using DNS with IoT is helpful for the IoT environment, it should be done carefully. The ability of IoT devices to reach DNS servers poses significant risks to the DNS infrastructure. This includes public DNS servers used on the Internet and private DNS setups used in isolated networks. Given the importance of DNS, these risks should not be overlooked.

The risks of using DNS with IoT could be summarized as follows:

- Complex coding at the IoT layer: The improper design of some IoT devices increases the probability of making simple mistakes when configuring them, but that could lead to DDoS (Distributed Denial-of-Service) attacks [14,15].
- DDoS attacks: The large number of IoT devices, coupled with their security vulnerabilities, allows for DDoS attacks of increased size and complexity against Internet infrastructure, which includes the DNS [14,15]. The Mirai botnet DDoS attack against the DYN DNS service provider had an unprecedented strength at the time, reaching 1.2 Tbps [12,13]. Such attacks are launched from several hundred thousand IoT devices that the attackers control. Having many devices under the attackers’ control makes countering the attack harder with traditional filtering based on IP addresses. This allows such DDoS attacks to last for extended durations.
- DDoS amplification: These attacks, also known as reflection attacks, depend on open resolvers’ response to a query which is usually larger than the original query. Adversaries might abuse this by sending several DNS queries but using the victim’s spoofed IP address as a source for these queries. The servers then respond and send responses to the victim’s machine. The massive load received by the victim could overload its memory and CPU and put it out of service [15].
- Recent DNS vulnerability: A vulnerability in several popular TCP/IP stacks used in some IT and IoT firmware was discovered in 2021. It was referred to as ‘Name: Wreck’, and it allows the devices to be used for remote code execution and denial of service attacks [15].

## 9. Discussion

This survey aimed to investigate the potential benefits of using DNS to address the IoT environments’ challenges. As illustrated in Figure 1, IoT technologies face several challenges. The scale at which IoT is integrated into daily life and the prediction of larger-scale deployments in the future demand that its challenges be scrutinized and alleviated one by one.

The constrained nature of IoT devices is one of its major limitations and simultaneously helped IoT spread. The constrained IoT devices are deprived of state-of-the-art protocols. These protocols are crafted for much more powerful devices on the Internet, such as personal computers, i.e., devices with enough processing power and memory. IoT devices, on the other hand, settle for inferior protocols, especially in terms of security, which jeopardizes data sent from and received by these devices. However, the processing, memory, and power constraints of IoT devices, even though they denied using state-of-the-art protocols, helped to spread the adoption of IoT technology due to their affordability and accessibility. DNS and its constrained-friendly extensions designed specifically for IoT could help IoT devices take advantage of modern tools adapted to their constraints.

The diverse IoT technologies that are mostly isolated from one another make it difficult to create a unified system for naming and identifying IoT devices. This is due to each manufacturer’s diverse communication and data representation protocols. Many of these protocols are proprietary and are not readily available for use by other technologies. The DNS, the Internet’s naming system, is the natural candidate to solve this issue. The well-established DNS infrastructure encourages using it to overcome the IoT identification problem. As discussed in Section 8, many initiatives already rely on DNS for IoT name resolution. However, a global solution is yet to be found.

Close to the identification challenge, there are significant interoperability issues between IoT technologies. These technologies are run by independent authorities, have their own standards, and are not compatible with one another. The IoT environment is thus fragmented and made up of independent vertical silos where one technology cannot communicate with the others due to the lack of standardization. This is crucial for the future of IoT, as having more interoperability between most, if not all, IoT technologies helps transform IoT into a global network. Furthermore, the lack of interoperability in IoT slows down research and development efforts supporting IoT, as different technologies disperse the research efforts, making progress more individualized to each technology rather than for the whole IoT environment. As a globally distributed infrastructure, the DNS has already significantly transformed the Internet into a cohesive network. It can continue to play a role in developing the IoT environment, turning it from independent silos to a more interconnected network.

Finally, regarding the security challenges of IoT, addressing these challenges becomes even more critical when we inspect the nature of the data that pass through such networks. The data that pass through IoT networks are mostly sensitive due to the diversity of IoT applications, from smart homes to medical and industrial applications. On the one hand, the ambitious calls to connect everything promises a prosperous and more comfortable future where additional everyday objects are connected and easily managed. On the other hand, it means higher stakes, as more data shared over the network means a higher risk of security breaches. DNS can contribute immensely to improving the security of IoT communications. DNSSEC, which is being adopted progressively in today’s Internet, can be used to ensure the integrity of DNS responses received by IoT devices. This helps prevent DNS cache poisoning attacks, spoofing attacks, and other DNS-related cyber threats. DNSSEC is essential for ensuring DNS response integrity, and its adoption is critical for enhancing the security of both IoT and regular Internet communications.

Moreover, to ensure privacy, DNS offers several extensions, some already standardized, like DoT and DoH, but they still need to be more widely adopted. DNS could also be adapted to constrained IoT, as with DoC. Therefore, to save IoT networks from being the weakest security link, using DNS and its security extensions and protocols is paramount.

## 10. Conclusions and Future Outlook

DNS is a valuable tool that has proved its worth as a pillar of today’s Internet. If used in IoT environments, it promises to address many of these environments’ challenges. Users, manufacturers, and administrators of IoT networks could reap huge benefits from exploiting the potential of DNS. As discussed in this survey, IoT faces several challenges related to device limitations and constraints, lack of standards, security issues, and interoperability problems. However, DNS can effectively address these challenges, enabling the widespread adoption and integration of IoT. DNS is flexible enough to be adapted to constrained devices, as seen with its extensions tailored to such devices as DNS over CoAP (DoC), which allows IoT technologies to benefit from its power. DNS is also highly scalable and can accommodate large numbers of devices, making it well-suited for the rapidly growing IoT landscape. Additionally, it can be beneficial when it comes to IoT identification. Moreover, DNS provides a universal standard for addressing devices, which could help overcome some of the interoperability issues of IoT environments and improve efficiency and reliability in IoT systems. Finally, DNS’s security extensions and ability to adapt to constrained IoT make it instrumental in ensuring that IoT systems are secure. However, it is essential to exercise caution when using DNS in IoT environments, and our survey highlighted the potential risks that IoT could pose to DNS infrastructure.

Looking towards the future, DNS is expected to integrate more into IoT environments. Some interesting domains to look into include having a global naming system similar to domain names for constrained IoT devices that preserves the privacy of IoT data. Another area of research could be the development of new DNS extensions designed for IoT environments. For example, there could be extensions that allow for more efficient and secure resolution of device names. Furthermore, location-based services and personalized content delivery could be some of the services DNS could provide to IoT technologies. In the context of Social IoT (SIoT) [148], which is a concept that aims to create a more human-centric IoT by allowing more interactions between humans and devices, DNS could play a fundamental role in facilitating connecting IoT devices to social media platforms and autoconfiguring their profiles. In addition, blockchain technologies’ increased security, transparency, and decentralization can benefit IoT applications. On the one hand, Distributed Sensor Networks (DSNs) relying on blockchain technologies can highly benefit from decentralized data storage, secure data transmission, transparency, and scalability offered by blockchains. On the other hand, DNS could also profit from blockchains, and this could provide a secure, decentralized name resolution and registration system [149,150]. Finally, IoT edge computing could benefit from DNS by leveraging DNS to discover and communicate with nearby edge computing resources.

## Figures and Tables

**Table 1:** List of abbreviations.

Abbreviation	Definition	Abbreviation	Definition
IoT	Internet of Things	DNS	Domain Name System
IP	Internet Protocol	TCP	Transmission Control Protocol
RFID	Radio-Frequency Identification	IoMT	Internet of Medical Things
IIoT	Industrial Internet of Things	RFC	Request for Comments
CoAP	Constrained Application Protocol	UDP	User Datagram Protocol
DOI	Digital Object Identifier	EPC	Electronic Product Code
URL	Uniform Resource Locator	URI	Universal Resource Identifier
ONS	Object Name Service	URN	Uniform Resource Names
MAC	Media Access Control	OID	Object Identifier
IETF	Internet Engineering Task Force	ORS	OID Resolution System
TLD	Top Level Domain	RR	Resource Record
DANE	DNS-Based Authentication of Named Entities	QNAME	Query Name
DDoS	Distributed Denial of Service	DNSSEC	DNS Security Extensions
DoH	DNS-over-HTTPS	DoT	DNS-over-TLS

**Table 2:** Classes of constrained devices (KiB = 1024 bytes) [56].

Name	Data Size (e.g., RAM)	Code Size (e.g., Flash)
Class 0, C0	≪10 KiB	≪100 KiB
Class 1, C1	∼10 KiB	∼100 KiB
Class 2, C2	∼50 KiB	∼250 KiB

**Table 3:** Taxonomy of IoT identifiers.

Identifier Category	Function	Use Case
Objects/Things Identifiers [59,60]	Used to identify the entity of interest, which could be physical or virtual	Sensors, machines, humans, merchandise (physical). Data, files, metadata (virtual)
Communication Identifiers [59,60]	Used to identify Things in the scope of communicating with other devices, including Internet-based communications.	IP address, MAC address, E.164
Application and Service Identifiers [59,60]	Used to identify applications/services used in the scope of IoT applications.	URL, URI, identifiers for different services on a single platform
User Identifier [60]	Used to identify physical or virtual objects that interact with IoT devices on the Internet.	ID for humans/animals (physical). ID for software applications interacting with IoT devices (virtual)
Data Identifier [60]	Used to identify data instances and datatypes	Digital Twin, stored sensor measurements
Location Identifier [60]	Used to specify a location within a geographical region	Coordinates, postal codes
Protocol Identifier [60]	Used to identify protocols so that, for example, layers within a communication stack can identify what protocols are being used by other layers	Ethertype

**Table 4:** Requirements of IoT Identifiers.

Requirement	Definition
Identify anything physical or virtual [61]	The identifier should be able to identify any physical or virtual thing as it is required that any physical or virtual thing can be connected to network infrastructure, which implies the necessity of having an identifier.
Communication between things [61]	Connection between things using identifiers, regardless if a particular thing needs to communicate or not, should be guaranteed.
Networking technology independence [61]	Identifiers should be independent of the underlying network technology used by the thing they identify.
Uniqueness [60]	Uniqueness is required within the specific application context. If a larger scope is needed where identifiers are no longer unique, a replacement or an extension of the identification scheme is always necessary to guarantee the identifier’s uniqueness.
Security and Privacy [60]	Identifiers used should be privacy- and personal-information-preserving. Ideally, identifiers should not leak information about the entity they define. The identifier by itself should not reveal, based on its structure, information about the identified thing.
Scalability [60]	The identification scheme should be able to accommodate the increasing number of identification-needing things in the future.
Interoperability [60]	Even if a single identification scheme does not exist, the existing and any newly proposed ones should account for interoperability between different schemes.

**Table 5:** Summary of DNS use cases in IoT environments.

DNS for Constrained IoT	DNS for IoT Name Resolution
QNAME minimization [114,115] and using elliptical curves [119] DNS over CoAP (DoC) [57,120] DNS over Datagram Transport Layer Security (DTLS) [121]	Object Name Service (ONS) [11] OID Resolution System (ORS) [68] IoT name resolution [122,123] Finding and localizing IoT devices [124,125] Device autoconfiguration [126,127,128] Device identification [129] IoT roaming [130]
**DNS for IoT Security**	**DNS for IoT Interoperability**
Secure IoT name resolution using DNS’s security extensions (see Section 7.3/Section 7.4). PKI for IoT [118,130] Authentication for device autoconfiguration [131,132] Malicious activity detection through DNS traffic analysis [133,134]	Overlay mechanisms for IoT naming [11,64,68,135] PKI that encourages IoT Interoperability using DNS and its security extensions [130] Interoperable service discovery using mDNS/DNS-SD [136,137,138]

## Data Availability

Data will be made available upon request.
